# Neural regulation of slow waves and phasic contractions in the distal stomach: a mathematical model

**DOI:** 10.1088/1741-2552/ad1610

**Published:** 2024-01-04

**Authors:** Omkar N Athavale, Recep Avci, Alys R Clark, Madeleine R Di Natale, Xiaokai Wang, John B Furness, Zhongming Liu, Leo K Cheng, Peng Du

**Affiliations:** 1 Auckland Bioengineering Institute, University of Auckland, Auckland, New Zealand; 2 Florey Institute of Neuroscience and Mental Health, Parkville, VIC, Australia; 3 Department of Anatomy & Physiology, University of Melbourne, Parkville, VIC, Australia; 4 Department of Biomedical Engineering, University of Michigan, Ann Arbor, MI, United States of America

**Keywords:** mathematical modelling, gastric motility, autonomic neuroscience, gastric electrophysiology

## Abstract

*Objective.* Neural regulation of gastric motility occurs partly through the regulation of gastric bioelectrical slow waves (SWs) and phasic contractions. The interaction of the tissues and organs involved in this regulatory process is complex. We sought to infer the relative importance of cellular mechanisms in inhibitory neural regulation of the stomach by enteric neurons and the interaction of inhibitory and excitatory electrical field stimulation. *Approach.* A novel mathematical model of gastric motility regulation by enteric neurons was developed and scenarios were simulated to determine the mechanisms through which enteric neural influence is exerted. This model was coupled to revised and extended electrophysiological models of gastric SWs and smooth muscle cells (SMCs). *Main results.* The mathematical model predicted that regulation of contractile apparatus sensitivity to intracellular calcium in the SMC was the major inhibition mechanism of active tension development, and that the effect on SW amplitude depended on the inhibition of non-specific cation currents more than the inhibition of calcium-activated chloride current (k_iNSCC_ = 0.77 vs k_iAno1_ = 0.33). The model predicted that the interaction between inhibitory and excitatory neural regulation, when applied with simultaneous and equal intensity, resulted in an inhibition of contraction amplitude almost equivalent to that of inhibitory stimulation (79% vs 77% decrease), while the effect on frequency was overall excitatory, though less than excitatory stimulation alone (66% vs 47% increase). *Significance.* The mathematical model predicts the effects of inhibitory and excitatory enteric neural stimulation on gastric motility function, as well as the effects when inhibitory and excitatory enteric neural stimulation interact. Incorporation of the model into organ-level simulations will provide insights regarding pathological mechanisms that underpin gastric functional disorders, and allow for *in silico* testing of the effects of clinical neuromodulation protocols for the treatment of these disorders.

## 1. Introduction

Gastric digestion is facilitated by coordinated contractions of gastric smooth muscles, which are governed by a range of regulatory mechanisms. These include rhythmic bioelectrical activity, named gastric slow waves (SWs), as well as neural and humoral factors [1, 2]. At the cellular level, SWs are generated and propagated by a network of excitable pacemaker cells called interstitial cells of Cajal (ICC), which are situated both between and within layers of gastric smooth muscles [3]. In conjunction with innervation from enteric neurons, SWs generated by ICC govern the phasic contractions of gastric smooth muscle cells (SMCs). Populations of excitatory and inhibitory enteric motor neurons release neurotransmitters at both ICC and SMC to regulate gastrointestinal motility [4, 5]. We postulate that an enhanced physiological understanding of the neural regulation of gastric motility will improve neuromodulation therapy for gastric disease, as has occurred in neuromodulation-based treatment of cardiac arrhythmogenesis and overactive bladder among other diseases [6, 7].

For clarity, throughout this work, SW refers to rhythmic electrical events, while phasic contraction refers to the contractions of gastric smooth muscles associated with SWs. ICC intrinsically undergo rhythmic depolarisations giving rise to SWs, while also being electrically connected in a syncytium [4]. ICC can be excited by each other, such that the region with the highest intrinsic frequency, known as the pacemaker region, entrains SWs across the entire stomach, thereby maintaining a sustained, regular propagation pattern. In large monogastric mammals, SWs are observed to emerge in the corpus along the greater curvature and form circumferential bands propagating in an antegrade direction towards the pyloric sphincter [8–11]. In other monogastric species, such as rodents, the exact location of the pacemaker region differs, but distinct functional regions related to motility remain evident [12].

Neural regulation of gastric motility is important, and its roles vary across the stomach [13]. Several neurotransmitters have been identified; acetylcholine (ACh) and tachykinins (TK) have roles in excitatory neuromuscular transmission, while nitric oxide (NO) and purinergic neurotransmission have major roles in the inhibitory pathway [14]. Neurotransmitters influence the frequency and amplitude of SWs and the underlying tonic state of the stomach, thereby affecting gastric volume, mixing, and emptying. The stomach is primarily innervated by the vagus nerve, forming a part of the brain-gut axis [2]. Pre-enteric, vagal efferent neurons innervate enteric nervous system (ENS) neurons at ganglia in the myenteric plexus, between the longitudinal and circular smooth muscle layers, but do not directly innervate the muscle [14, 15]. ENS neurons directly innervate ICC, SMC, and other ENS neurons, while, in contrast, efferent vagal neurons innervate ENS neurons but not ICC or SMC [2].

While experimental work has identified a variety of candidate mechanisms enacting post-junctional neurotransduction, the relative importance of each mechanism in relation to gastric motility control is yet to be clarified. Complexity arises from the interaction and targeting of both ICC and SMC by enteric neurons, and the variety of expressed channels in both these cell types that are involved in post-junctional events. Existing models of gastrointestinal electrophysiology or motility focus on either ICC [16–19] or SMC [20–22]. However, none of these models have incorporated the effects of neural regulation on the properties of the phasic contractions which enable gastric motility. There exists scope to examine the influence that putative mechanisms of inhibitory neural regulation, termed ‘effector components’, have on SWs and phasic contractions in relation to experimental observations. Furthermore, a modelling study can predict the interaction between inhibitory and excitatory neural regulation. The model developed herein addresses this knowledge gap by incorporating the effects of efferent neural regulation on gastric ICC and gastric SMC. It models how cell level mechanisms interact to control SW activity. This improvement enables the computational study of how one portion of the neural regulation pathways to the stomach control gastric motility [1].

Inhibitory neural regulation via nitrergic and purinergic pathways targets both ICC and SMC in the distal stomach. The nitrergic pathway acts through the activation of cyclic guanosine monophosphate (cGMP) intracellular signalling pathways by NO. Potential targets in ICC include Ano1 [5], transient receptor potential melastatin (TRPM) [23–25], and large-conductance K^+^ (BK) channels [26, 27] though the effects at BK are better documented in intestinal SMC rather than gastric ICC [28, 29]. TRPM channels are non-specific cationic conductance channels (NSCCs). Purinergic neurotransduction via apamin-sensitive small-conductance K^+^ (SK) channels has also been identified in the distal stomach [30], likely via fibroblast-like (PDGFR*α* ^+^) cells conducting to SMC [31, 32]. By observation of fast and slow inhibitory junction potentials (IJPs) it was deduced that ICC are not involved in the purinergic pathway [33]. Additionally, the intracellular Ca^2+^ sensitivity of the SMC contractile apparatus is affected by nitrergic stimulation [34].

Excitatory cholinergic neural regulation affects the frequency of SWs in ICC. Effects on the ICC SW arise from increased IP_3_ production of due to muscarinic activation by acetylcholine (ACh). This causes increased Ca^2+^ release from intracellular stores, through the IP_3_-activated Ca^2+^ channel (IP_3_R), thereby resulting in a higher frequency of SW activation [35]. Experiments have shown that tachykinins, which may be co-transmitted with ACh, impart an additional excitatory effect on SW frequency at stimulation frequencies of 10 Hz or higher [36].

A mathematical model was developed to elucidate the cellular mechanisms for enteric neural regulation of gastric SWs and associated phasic contractions. Five key cellular mechanisms were incorporated for inhibitory and excitatory neural regulation of ICC and SMC, as well as their interactions and effects on gastric motility. Four of these were inhibitory regulation mechanisms via NO modulation of Ano1, NSCC, and SK channels, and inhibitory regulation of SMC calcium sensitivity. The fifth was a mechanism of the excitatory regulation of IP_3_. The model was parameterised to reflect the properties of the rat distal stomach, for alignment with literature on rat gastric neural regulation [36, 37]. Our model allows gastric motility to be simulated under varied enteric motor neuron stimulation frequency and assess the contribution of different post-junctional cellular mechanisms.

## 2. Methods

**Figure 1.**
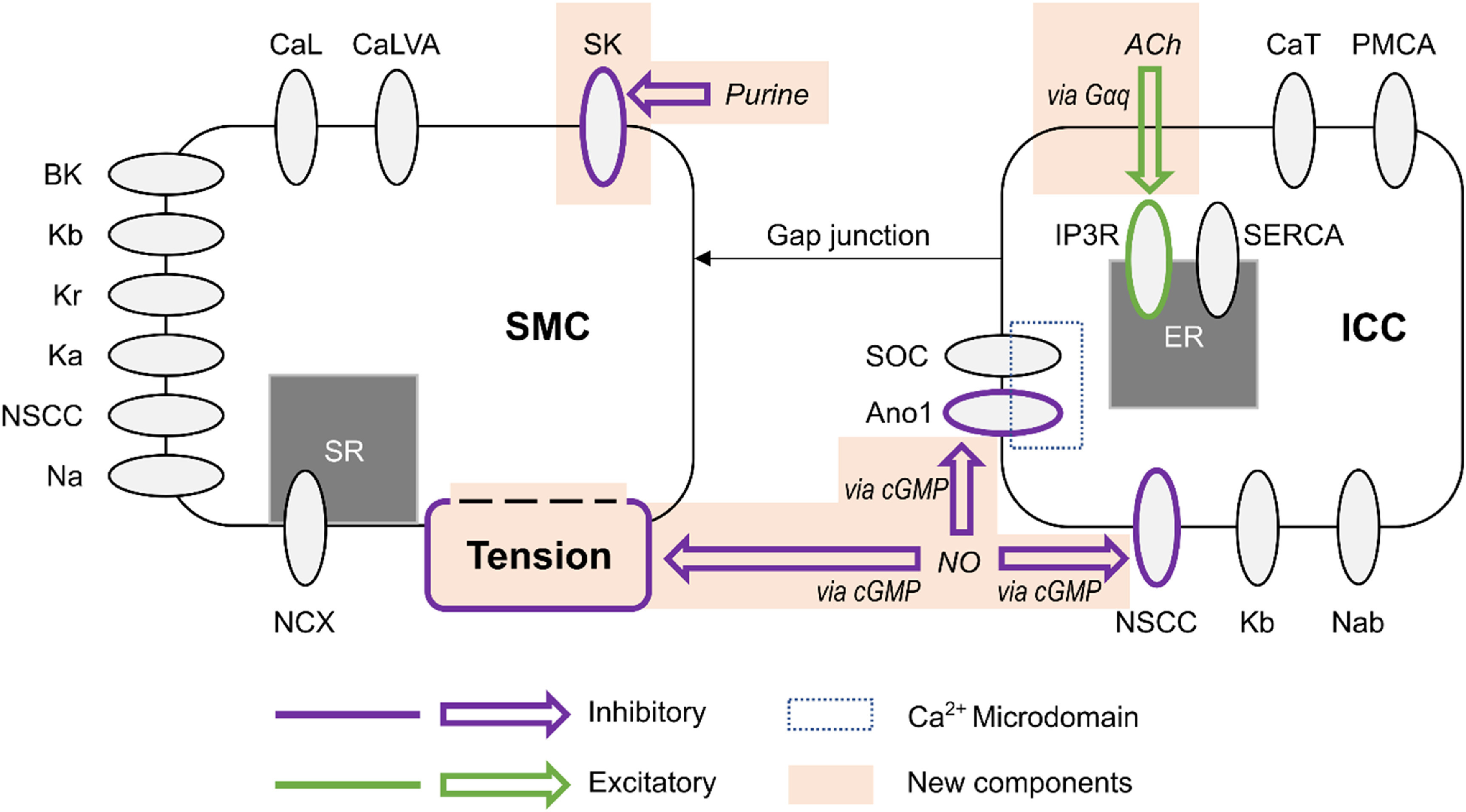
Schematic representation of the coupled electrophysiology model of a distal gastric interstitial cell of Cajal (ICC) and gastric smooth muscle cell (SMC), illustrating the modelled neural regulation pathways. The gastric SMC model developed by Corrias and Buist [20] was coupled to a reparameterised model of ICC originally developed by Lees-Green *et al* [18]. The components shaded in orange are novel additions formulated in the present work. Inhibitory effector components are outlined in purple and excitatory effector components are outlined in green. CaL: L-type calcium channel, CaLVA: T-type calcium channel (see also CaT in the ICC model), SK: small conductance potassium channel, BK: large conductance potassium channel, Kb: lumped background potassium current, Kr: delayed rectifying potassium channel, Ka: A-type voltage-gated potassium channel, NSCC: non-specific cationic conductance, Na: lumped sodium current, NCX: sodium-calcium exchanger, SR: sarcoplasmic reticulum, Gαq: G-protein α subunit, ACh: acetylcholine, CaT: T-type calcium channel (see also CaLVA in the SMC model), PMCA: plasma membrane calcium ATPase, IP3R: IP3-activated calcium channel, SERCA: sarcoendoplasmic reticulum calcium ATPase, SOC: store-operated calcium channel, Ano1: anoctamin-1 voltage-gated, calcium activated chloride channel, cGMP: cyclic guanosine monophosphate second messenger nucleotide, NO: nitric oxide, Nab: lumped background sodium current.

The proposed model comprises an electrophysiological model coupling ICC and SMC via gap junctions, an active tension model bridging electrophysiology and biomechanics, and a model simulating neural regulation through nitrergic, purinergic, and cholinergic pathways, as illustrated in figure 1.

The Lees-Green small intestinal ICC model [18] was reparameterised for gastric SWs. Within this model, the Ano1, NSCC, and IP_3_R components (figure 1) were modified to incorporate enteric neural regulation. The Corrias and Buist [20] gastric SMC model was used to simulate intracellular SMC Ca^2+^ concentration and new SK and active tension components were formulated to extend this model and incorporate enteric neural regulation of SMC.

### 2.1. Interstitial cell of Cajal model

The ICC model incorporates four ionic currents modelled by Hodgkin–Huxley formalisms with Ca^2+^ or voltage dependence: T-type Ca^2+^ channel (CaT); a lumped background K^+^ channel (Kb); background Na channel (Nab); and NSCC. Additionally, a model of store operated calcium entry is modelled within a Ca^2+^ microdomain for the Ca^2+^-dependent and voltage-dependent Ano1, Cl^−^ anion channel. Note that myenteric ICC (ICC-MY) and intramuscular ICC (ICC-IM) are modelled as a single entity.

The ICC model was modified to simulate the frequency of SW activity in distal stomach rather than the small intestine. Lees-Green *et al* adopted calcium dynamics from a model of murine airway SMC [38], which were themselves adapted from the de Young–Keizer model of single pool calcium dynamics [39]. As indicated in table 1, time constants for the IP_3_R channel were modified to simulate the SW frequency of the rat stomach. Additionally, to maintain physiological calcium levels, the maximal rate of Ca^2+^ sequestration by sarcoendoplasmic reticulum calcium ATPase (SERCA) was increased. It was verified that the effect of Ano1 knockout, as simulated by the original ICC model, was maintained after making the specified changes to calcium dynamics. In the modified model, Ano1 knockout (modelled as Ano1 conductance set to 0 nS) results in small amplitude oscillations of ICC membrane potential (5 mV). Furthermore, *E*_Cl_ was set to −11 mV to more closely align with experimentally measured values in cultured murine myenteric ICC [40]. The role of a Ca^2+^-dependent chloride current (CaCC), such as that facilitated by Ano1, in maintaining the plateau potential of the ICC SW was identified by Kito *et al* [41]. A decrease in Cl^−^ conductance has been proposed as a mechanism for nitrergic inhibition in ICC due to its observed involvement in the IJP [42].

**Table 1.** Parameters modified in the Lees-Green *et al* (2014) ICC model to adjust the Ca^2+^ oscillation frequency. These parameters are based on the de Young–Keizer model [39] of single pool calcium dynamics. Note that K_i_ = k_i−_/k_i_ in equations (22), (24) and (25). SERCA: sarcoendoplasmic reticulum calcium ATPase, IP_3_: inositol triphosphate.

Variable description		Variable	Value
Receptor binding (*µ*M^−1^ s^−1^)	IP_3_	*k*_1_	500
	Ca^2+^ inhibition	*k*_2_	0.25
	IP_3_	*k*_3_	500
	Ca^2+^ inhibition	*k*_4_	0.25
	Ca^2+^ activation	*k*_5_	25
Receptor unbinding (s^−1^)	IP_3_	*k*_1-_	65
	Ca^2+^ inhibition	*k*_2-_	0.2625
	IP_3_	*k*_3-_	471.5
	Ca^2+^ inhibition	k_4-_	0.03625
	Ca^2+^ activation	*k*_5-_	2.05
Maximal SERCA rate (*µ*M s^−1^)		*V*_e_	160

### 2.2. Smooth muscle cell (SMC) model

The SMC model includes a variety of membrane currents to model gastric SMC electrophysiology, with SW events driven by current injected via a gap junction (figure 1). The original implementation included a prescribed ICC membrane potential to set the gap junction current. In the present work, this prescribed ICC potential was instead directly coupled to the ICC model via a gap junction current, *I*_couple_,

\begin{equation*}{I_{{\text{couple}}}} = {g_{{\text{couple}}}} \cdot \left( {{V_{{\text{ICC}}}} - {V_{{\text{SMC}}}}} \right)\end{equation*}

where *g*_couple_ is the gap junction conductance, *V*_ICC_ is the ICC membrane potential, and *V*_SMC_ is the SMC membrane potential.

### 2.3. Active tension model

A Hill-type relationship was used to model the Ca^2+^-force relationship,

\begin{equation*}T = {T_0} \cdot \frac{{{\text{C}}{{\text{a}}_{\text{i}}}^{\text{h}}}}{{{\text{C}}{{\text{a}}_{\text{i}}}^{\text{h}} + {\text{C}}{{\text{a}}_{{\text{50}}}}^{\text{h}}}}\end{equation*}

where *T* is the active tension, Ca_i_ denotes the intracellular calcium concentration of the SMC model, *T*_0_ is the maximal tension, *h* denotes the Hill sensitivity coefficient, and Ca_50_ denotes the concentration of cytosolic Ca^2+^ at half maximal tension. Ca_50_ was modified by inhibitory neural regulation (see equation (16)) and the values for *T*_0_ and *h* (313 kPa and 3.4, respectively) were obtained from isometric tension generation measurements using skinned smooth muscle strips from guinea pig taenia coli as reported by Arner [43]. Mechano-sensitive mechanisms were not incorporated into this model.

### 2.4. Neural regulation model

Efferent neural regulation by the stimulation of intrinsic motor neurons was included in this model, but reflex circuitry was not considered. Neural stimulation was modelled by variables for inhibitory and excitatory stimulation *f*_i_ and *f*_e_. These variables represent the frequency of electrical field stimulation between 0 and 10 Hz for inhibitory nitrergic and purinergic stimulation, and excitatory cholinergic stimulation, respectively. The stimulation of intrinsic motor neurons is modelled as having a steady-state response, in concert with results from *in vitro* experiments where electrical field stimulation pulses of 0.1 ms pulse width were applied at a fixed frequency [36, 37]. Terms were formulated to model inhibitory effects (*S*_iX_) at the effector components, Ano1 (*S*_iAno1_), NSCC (*S*_iNSCC_), Ca^2+^ sensitivity (*S*_iCa50_), and SK (*S*_iSK_),

\begin{equation*}{S_{{\text{iAno1}}}} = {k_{{\text{iAno1}}}} \cdot {w_{{\text{iICC}}}}\end{equation*}

\begin{equation*}{S_{{\text{iNSCC}}}} = {k_{{\text{iNSCC}}}} \cdot {w_{{\text{iICC}}}}\end{equation*}

\begin{equation*}{S_{{\text{iCa50}}}} = {k_{{\text{iCa50}}}} \cdot {w_{{\text{iSMC}}}}\end{equation*}

\begin{equation*}{S_{{\text{iSK}}}} = {k_{{\text{iSK}}}} \cdot {w_{{\text{iSMC}}}}\end{equation*}

where *k*_iAno1_, *k*_iNSCC_, *k*_iCa50,_ and *k*_iSK_ are inhibitory effector component weighting terms. The excitatory stimulation effect, *S*_e_, affecting IP_3_ concentration was modelled by,

\begin{equation*}{S_\text{e}} = {k_{\text{eIP3}{\text{ }}}} \cdot {w_\text{e}}\end{equation*}

where *k*_eIP3_ is an excitatory effector component weighting term.

Scaling terms *w*_iICC_ and *w*_iSMC_, for ICC and SMC based inhibitory effector components respectively, and w_e_ for the excitatory effector component were calculated as functions of inhibitory (*f*_i_) and excitatory (*f*_e_) stimulation frequency

\begin{equation*}{w_{{\text{iICC}}}} = \frac{{1 - \frac{{{e^{ - {p_{{\text{iICC}}}}{f_{\text{i}}}}}}}{{{f_{\max }}}}}}{{1 - {e^{ - {p_{{\text{iICC}}}}}}}}\end{equation*}

\begin{equation*}{w_{{\text{iSMC}}}} = \frac{{1 - {e^{ - \frac{{{p_{{\text{iSMC}}}}{f_{\text{i}}}}}{{{f_{\max }}}}}}}}{{1 - {e^{ - {p_{{\text{iSMC}}}}}}}}\end{equation*}

\begin{equation*}{w_{\text{e}}} = \frac{{1 - {e^{ - \frac{{{p_{\text{e}}}{f_{\text{e}}}}}{{{f_{\max }}}}}}}}{{1 - {e^{ - {p_{\text{e}}}}}}}\end{equation*}

where *f*_max_ is the maximum modelled input stimulation frequency fitted from experimental values [36, 37]. For fitting purposes, the frequency of electrical field stimulation during continuous stimulation on the order of minutes was used, in conjunction with data from pharmacological stimulation matched in tissue response to electrical field stimulation. The parameters, *p*_iICC_, *p*_iSMC_, and *p*_e_ determine the non-linearity of the relationship between stimulation frequency and the effect of stimulation.

The value of *k*_eIP3_ and *p*_e_ were selected to reproduce the results from Forrest *et al* [36]. Values for the inhibitory effector components (*k*_iAno1_, *k*_iNSCC_, *k*_iCa50,_ and *k*_iSK_) were selected by an optimisation procedure (see section 2.6). Fitting of the excitatory pathway parameters was excluded from the optimisation procedure because the experimental data for fitting inhibitory pathway parameters used pharmacological methods to block cholinergic neurotransmission. Conversely, data used to fit the excitatory pathway parameters was obtained under pharmacological block of nitrergic neurotransmission, so the excitatory parameters could be estimated in isolation from the inhibitory parameters.

#### 2.4.1. Nitrergic pathway

Inhibition of the Ano1 channel by cGMP occurs due to a decreased current during Ca^2+^-dependent activation. To model this, an inhibition term was formulated as

\begin{equation*}{h_{{\text{Ano}}1}} = 1 - \frac{{{S_{{\text{iAno1}}}}}}{{1 + {{\left( {d_{{\text{Ano}}1}^{^{^{\prime}}}} \right)}^2}}}\end{equation*}

where *h*_Ano1_ is the Ano1 inhibition term, *d*′_Ano1_ is the time derivative of the Ano1 gating variable, *d*_Ano1_. This term inhibits activation during the calcium-activated plateau phase of the Ano1 current. Hence, the modified Ano1 current, *I*_Ano1_, in the ICC model was given by

\begin{equation*}{I_{{\text{Ano1}}}} = {g_{{\text{Ano1}}}} \cdot \left( {{d_{{\text{Ano1}}}} - {h_{{\text{Ano1}}}}} \right) \cdot \left( {{V_{{\text{ICC}}}} - {E_{{\text{Cl}}}}} \right)\end{equation*}

where *g*_Ano1_ is the maximal conductance of the Ano1 channel, *V*_ICC_ is the ICC membrane potential and *E*_Cl_ is the reversal potential for Cl^−^.

Additionally, the role of NSCC as a potential effector of nitrergic stimulation was investigated by modulating the channel conductance, *g*_NSCC_, which is applied to calculate the NSCC channel current, *I*_NSCC_, defined by the following equations:

\begin{equation*}{I_{{\text{NSCC}}}} = {g_{{\text{NSCC}}}} \cdot {P_{{\text{NSCC}}}} \cdot \left( {{V_{{\text{ICC}}}} - {E_{{\text{NSCC}}}}} \right)\end{equation*}

\begin{equation*}{g_{{\text{NSCC}}}} = {g_0} \cdot \left( {1 - {S_{{\text{iNSCC}}}}} \right)\end{equation*}

\begin{equation*}{P_{\text{NSCC}}} = \frac{{Ca_i^{{n_{\text{NSCC}}}}}}{{Ca_i^{{n_{\text{NSCC}}}} + Ca_{\text{NSCC}}^{{n_{\text{NSCC}}}}{\text{ }}}}\end{equation*}

where *P*_NSCC_ is a Ca^2+^-dependent gating variable, *E*_NSCC_ is the reversal potential, *g*_0_ is the maximum NSCC conductance, Ca_i_ is the intracellular calcium concentration, *n*_NSCC_ is the Hill coefficient, and Ca_NSCC_ is the half-maximal intracellular Ca^2+^ concentration for gating activation.

Additionally, the effect of nitrergic stimulation on Ca^2+^ sensitivity in SMC was also investigated, by shifting the Ca^2+^-force relationship modelled in equation (2), based on the level of inhibitory stimulation. Ca_50_ was calculated by

\begin{equation*}{\text{C}}{{\text{a}}_{50}} = {\text{C}}{{\text{a}}_0} \cdot \left( {1 + {S_{{\text{iCa50}}}}} \right)\end{equation*}

where the baseline calcium sensitivity, Ca_0_, was 0.56 *μ*M [43].

#### 2.4.2. Purinergic pathway

A new SK channel was added to the SMC model by adapting a model of ventricular myocyte SK channels developed by Kennedy *et al* [44]. The SK channel current was modelled by

\begin{equation*}{I_{{\text{SK}}}} = {g_{{\text{SK}}}} \cdot {d_{{\text{SK}}}} \cdot {S_{{\text{iSK}}}} \cdot \left( {{V_{{\text{SMC}}}} - {E_{\text{K}}}} \right)\end{equation*}

where *I*_SK_ is the membrane SK channel current, *g*_SK_ is the maximal channel conductance, *E*_K_ is the K^+^ reversal potential, and *d*_SK_ is a Ca^2+^-dependent gating variable given by

\begin{equation*}\frac{{{\text{d}}{d_{{\text{SK}}}}}}{{{\text{d}}t}} = \frac{{{d_{{\text{SK}}\infty }} - {d_{{\text{SK}}}}}}{{{\tau _{{\text{SK}}}}}}\end{equation*}

\begin{equation*}{\tau _{{\text{SK}}}} = \frac{1}{{0.047{\text{C}}{{\text{a}}_{\text{i}}} + 0.013}}\end{equation*}

\begin{equation*}{d_{{\text{SK}}\infty }} = \frac{{0.81{\text{C}}{{\text{a}}_{\text{i}}}}}{{{\text{Ca}}_{\text{i}}^{{n_{{\text{SK}}}}} + {\text{Ca}}_{{\text{SK}}}^{{n_{{\text{SK}}}}}}}\end{equation*}

where *d*_SK∞_ is the scaling gating constant, *τ*_SK_ is the gating time constant, *n*_SK_ is the Hill coefficient of the scaling constant, and Ca_SK_ is the half-maximal Ca^2+^ concentration of the scaling gating constant.

#### 2.4.3. Cholinergic pathway

Cholinergic stimulation was modelled by modification of the IP_3_ production rate in the ICC model. Intracellular IP_3_ production rate is given by

\begin{equation*}{\text{I}}{{\text{P}}_3} = {\text{IP}}{3_0} \cdot \left( {1 + {S_{{\text{eIP3}}}}} \right)\end{equation*}

where IP3_0_ is the baseline rate of IP_3_ generation. The IP_3_ production rate affects the following equations:

\begin{equation*}{P_{{\text{IPR}}}} = {\left( {\frac{{{\text{I}}{{\text{P}}_3} \cdot {\text{C}}{{\text{a}}_{\text{i}}} \cdot \left( {1 - y} \right)}}{{\left( {{\text{I}}{{\text{P}}_3} + {K_1}} \right) \cdot \left( {{\text{C}}{{\text{a}}_{\text{i}}} + {K_5}} \right)}}} \right)^3}\end{equation*}

\begin{equation*}{J_{{\text{IPR}}}} = \left({k_{{\text{IPR}}}}{P_{{\text{IPR}}}} + {J_{{\text{ER}}}}\right) \cdot \left( {{\text{C}}{{\text{a}}_{{\text{ER}}}} - {\text{C}}{{\text{a}}_{\text{i}}}} \right)\end{equation*}

\begin{equation*}{\phi _1} = \frac{{{\text{C}}{{\text{a}}_{\text{i}}} \cdot \left( {{k_4} \cdot {K_2} \cdot {K_1} + {k_2} \cdot {K_4} \cdot {\text{I}}{{\text{P}}_3}} \right)}}{{{K_4} \cdot {K_2} \cdot \left( {{K_1} + {K_3}} \right)}}\end{equation*}

\begin{equation*}{\phi _2} = \frac{{{k_2} \cdot {\text{I}}{{\text{P}}_3} + {k_4} \cdot {K_3}}}{{{K_3} + {\text{I}}{{\text{P}}_3}}}\end{equation*}

\begin{equation*}\frac{{{\text{d}}y}}{{{\text{d}}t}} = {\phi _1} \cdot \left( {1 - y} \right) - {\phi _2} \cdot y\end{equation*}

\begin{equation*}\frac{{{\text{dC}}{{\text{a}}_{\text{i}}}}}{{{\text{d}}t}} = {f_{\text{c}}} \cdot \left( {{J_{{\text{IPR}}}} - {J_{{\text{SERCA}}}} + {J_{{\text{SOC}}}} - {J_{{\text{PMCA}}}}} \right)\end{equation*}

where *J*_IPR_ is the calcium release flux due to IP_3_-activated Ca^2+^ channels (IP_3_Rs), *k*_IPR_ is a scaling constant, *P*_IPR_ is the Ca_i_ dependent gating variable for IP_3_R, *J*_ER_ is the leak rate, Ca_ER_ is the calcium concentration within the intracellular store, IP_3_ is the intracellular IP_3_ concentration, *y* is the binding variable for IP_3_ to IP_3_R, *ϕ*_1_ and *ϕ*_2_ are the binding and unbinding probability respectively for IP_3_ to IP_3_R, based on the rate constants *K*_1_ − *K*_5_ (table 1), *f*_c_ scales the intracellular calcium level, and *J*_x_ are the rates of calcium flux for the channels IP_3_R, SERCA, SOCE, and PMCA (figure 1).

### 2.5. Model simulation and metric quantification

The model was implemented in CellML, and solved using the ‘ode15s’, variable step, variable order integrator in MATLAB 2022b (The MathWorks, Natick, MA, USA). The absolute and relative tolerances were both 1 × 10^−6^ and the maximum step size was 1 ms. To confirm model stability a 300 s simulation was conducted. Following this, in every case where analysis was performed, the system was solved for 60 s to ensure a steady state oscillatory solution and analysis was performed on a further 120 s of simulation time. Unless specified, stimulation was applied for the entire simulation. Metrics were quantified to characterise the steady state oscillatory solution, frequency in cycles per minute (cpm), initial peak tension (kPa), and plateau tension (kPa). Additionally, initial peak depolarisation and plateau potential of SMC or ICC membrane potentials (mV) were also quantified as required.

For both membrane potential and active tension, the initial peak was defined as the maximum value during a 100 ms period after the peak positive rate of change. The amplitude values are given as a difference from baseline (resting membrane potential or baseline tension). The plateau value was defined as the mean value during which the smoothed absolute derivative (moving mean: 200 ms window) was less than 2.5 mV s^−1^ during a 3 s window after peak depolarisation. The derivative threshold and window size for identifying the plateau period were selected by inspecting the result for extreme cases of parameter values.

### 2.6. Parameter optimisation

To select parameter values for the four inhibitory weighting terms *k*_iAno1_, *k*_iNSCC_, *k*_iCa50_, and *k*_iSK_ and two non-linear scaling terms *p*_iICC_ and *p*_iSMC_, a constrained, non-linear optimisation problem was solved using the interior-point algorithm (‘fmincon’ in MATLAB 2022b) to minimise the objective function,

\begin{equation*}o = \mathop \sum \limits_{n = 1}^4 \left| {\frac{{M\left( {\vec z,{\text{ }}{f_n}} \right)}}{{{M_0}}} - E\left( {{f_n}} \right)} \right|\end{equation*}

where *M* is a function representing the simulated value of the optimised quantity, while *E* is a function representing the percentage change in quantity at a stimulation frequency, *f_n_*, as determined by experimental work. The parameter values for the simulation, were captured in the vector $\vec z$. *M*_0_ is the simulated plateau tension amplitude for *f*_i_ = 0 Hz (i.e. no stimulation) with input parameters $\vec z$.

The fitting procedure was performed separately for parameters in the ICC (Step 1) and SMC (Step 2) models, as described in table 2. First, the parameters in the ICC model were fit to data for the membrane potential change during inhibitory stimulation. Then, with the ICC parameters fixed, the parameters for the Ca^2+^ sensitivity, SK channel, and *p*_iSMC_ were fit to experimental data measuring the change in tension during phasic contractions under inhibitory stimulation. The optimisation procedure was performed 48 times using initialisations randomly sampled from a Sobol set of the feasible parameter space (see results section). Using 48 initialisations improved coverage of the parameter space and allowed for the identification of potential local minima. The median value of the solutions to Step 1 had a marginal change of less than ±2% in all parameters when more than approximately 24 initialisations were used after randomly permuting the initialisation sequence 100 times. Therefore, twice this number, 48, was deemed to be a sufficient number of initialisations. After 48 initialisations the marginal change in median value was less than 0.35% for all three ICC parameters. The values of *k*_eIP3_ and *p*_e_ were selected based on experimental measurements of SW frequency change during cholinergic stimulation (see section 3).

**Table 2.** Steps in the parameter optimisation procedure.

	Step 1 (ICC)	Step 2 (SMC)
Parameters optimised	${{\text{k}}_{{\text{iAno}}1}},{\text{ }}{{\text{k}}_{{\text{iNSCC}}}},{\text{ }}{{\text{p}}_{{\text{iICC}}}}$	${{\text{k}}_{{\text{iCa}}50}},{\text{ }}{{\text{k}}_{{\text{iSK}}}},{\text{ }}{{\text{p}}_{{\text{iSMC}}}}$
Other parameters	${k_{{\text{iCa}}50}} = {k_{{\text{iSK}}}} = 0$, ${p_{{\text{iSMC}}}} = 1$	${{\text{k}}_{{\text{iAno}}1}},{\text{ }}{{\text{k}}_{{\text{iNSCC}}}},{\text{ }}{{\text{p}}_{{\text{iICC}}}}{\text{ }}$from the solution of Step 1 (see results section 3)
Experimental data	Figures 1(B) and 2(E) from Kim *et al* [37]	Figure 1(B) from Kim *et al* [37]
Quantity optimised	Slow wave plateau amplitude	Phasic contraction plateau amplitude
Constraints (further explanation in results)	$1.11{\text{ }}{{\text{k}}_{{\text{iAno}}1}} + {\text{ }}{{\text{k}}_{{\text{iNSCC}}}} \unicode{x2A7D} 1.41$, $0 &lt; {k_{{\text{iNSCC}}}} &lt; 1$, $0 &lt; {k_{{\text{iNSCC}}}} &lt; 1$	$0 &lt; {k_{{\text{iCa}}50}} &lt; 3$, $0 &lt; {k_{{\text{iSK}}}} &lt; 1$

### 2.7. Sensitivity analysis

The effect of perturbing the effector component weighting parameters (*k*_iAno1_, *k*_iNSCC_, *k*_iCa50_, and *k*_iSK_) between the 10 and 90th percentile of the optimisation procedure solution was investigated. Additionally, the excitatory component weighting (*k*_eIP3_) was also perturbed between the minimum and maximum estimates of the parameter value. The Saltelli method was used to obtain a representative, uniformly distributed sample of this parameter space [45] with 1024 sampled points per analysed variable. Sampling and analysis were performed in Python using the ‘SALib’ module, while simulations were run and results quantified in MATLAB as described in section 2.5 [46]. Maximal inhibitory and excitatory stimulation (*f*_i_ = *f*_e_ = 10 Hz) was set for each sampled parameter combination. The first-order Sobol sensitivity (*S*_1_) index and the total Sobol sensitivity (*S*_T_) index for a given variable *X_i_* were calculated as follows

\begin{equation*}{S_1} = \frac{{{V_{{X_i}}}\left( {{E_{{X_{\sim i}}}}\left( {Y{\text{|}}{X_i}} \right)} \right)}}{{V\left( Y \right)}}\end{equation*}

\begin{equation*}{S_{\text{T}}} = \frac{{{E_{{X_{\sim i}}}}\left( {{V_{{X_i}}}\left( {Y{\text{|}}{X_{\sim i}}} \right)} \right)}}{{V\left( Y \right)}}\end{equation*}

where *V* represents the variance, *E* represents the expected value, *X*_∼*i*_ is all variables except the *i*th variable, and *Y* is the metric value.

## 3. Results

**Figure 2.**
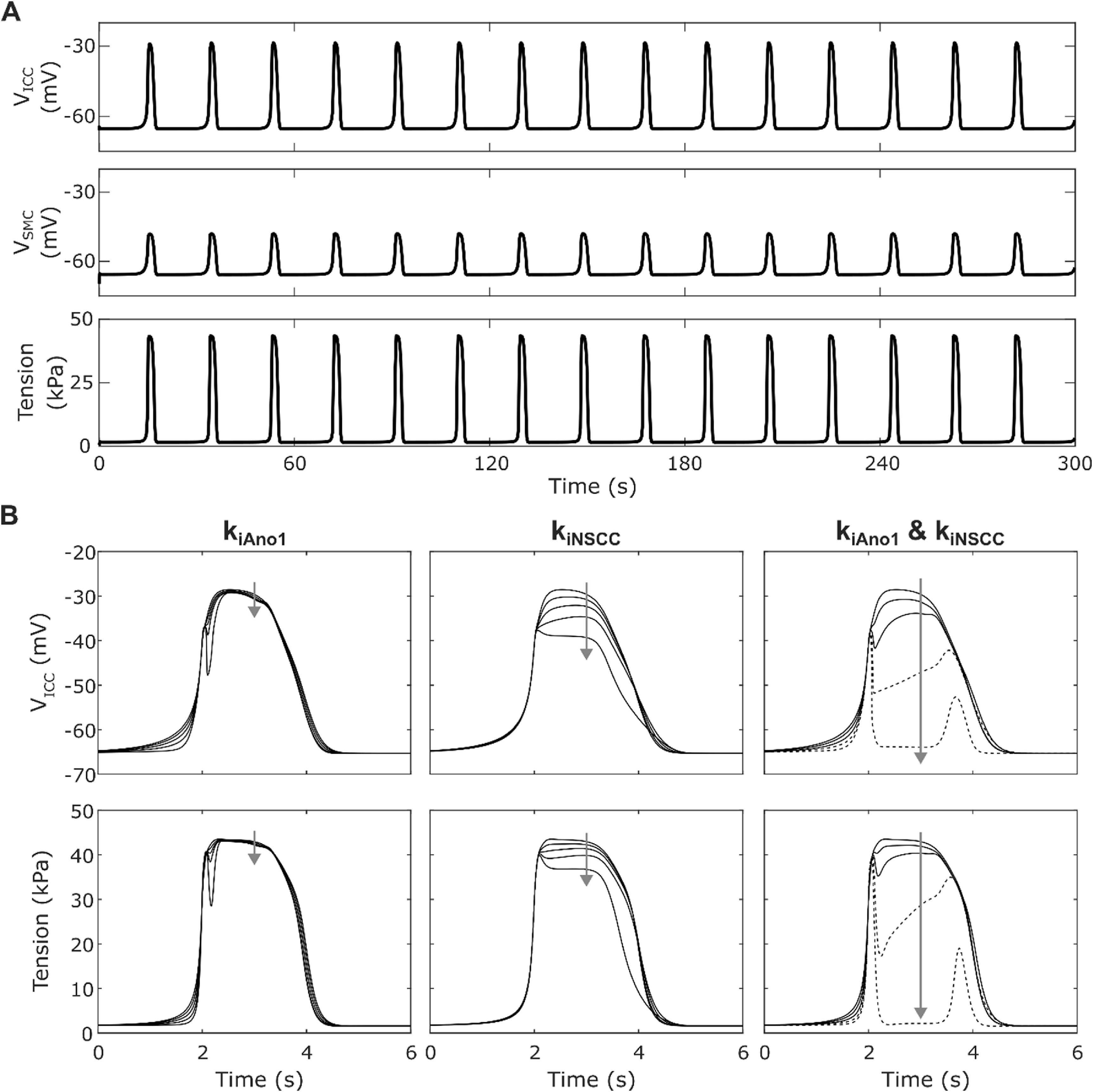
(A) Membrane potential for ICC (top), SMC (middle) and active tension (bottom) simulated with no inhibitory or excitatory stimulation over 300 s. The resting membrane potential was stable and slow wave frequency was consistent throughout the simulation. (B) ICC membrane potential (top) and active tension (bottom) during a single slow wave cycle with varying k_iAno1_ alone (left), k_iNSCC_ alone (centre), and k_iAno1_ and k_iNSCC_ together with k_iAno1_ = k_iNSCC_ (right) from 0 to 1 in 0.25 increments and f_i_ set to 10 Hz. Arrows indicate increasing weighting (k_iAno1_ and k_iNSCC_) values. Inhibition of Ano1 and NSCC alone does not give an appropriate decrease in plateau membrane depolarisation. Inhibition of both Ano1 plateau current and NSCC is required to yield a closer match to experimental data, but a complete block of both components (k_iAno1_ = k_iNSCC_ = 1) results in non-physiological behaviour as indicated by the broken lines.

Stable solutions were obtained for *V*_ICC_ and *V*_SMC_ with a self-oscillatory SW frequency of 3.2 cpm after simulating for 300 s beginning at the initial conditions, with the SW interval varying by no more than 3 ms. This resulted in phasic contractions at the same frequency (figure 2(A)). The resting membrane potential, initial peak depolarisation amplitude potential and plateau amplitude potential of each SW event was consistent over time for both the ICC (−65.3 mV, 26.2 mV, and 36.6 mV respectively) and SMC (−65.7 mV, 16.0 mV, and 17.6 mV respectively). The phasic contraction amplitude was also consistent over the 300 s window, with a basal tension of 1.6 kPa, an initial peak tension amplitude of 43.3 kPa and a plateau amplitude of 41.7 kPa. The resting membrane potentials and basal tension varied by less than 0.0001 mV and less than 0.0001 kPa respectively over a 300 s simulation. Similarly, over the same period the initial peak amplitude of *V*_ICC_, *V*_SMC_, and peak tension varied by 0.010 mV, 0.002 mV, and 0.007 kPa respectively. SW events were synchronised but not necessarily time-aligned between tension, SMC membrane potential, and ICC membrane potential.

Of the four inhibitory effector components, only two of these affect membrane currents in the ICC: Ano1 and NSCC. Varying the respective weightings of these components (*k*_iAno1_, *k*_iNSCC_) in the inhibitory stimulation pathway showed that when both components were non-zero, the effect on the ICC SW plateau was much greater than if only one was non-zero (figure 2(B)). However, the initial peak depolarisation was not affected by *k*_iAno1_ and *k*_iNSCC_. Furthermore, when both components had a high weighting, a non-physiological SW signal morphology exhibiting a second depolarisation resulted (figure 2(B) right). By performing a parameter sweep for the values of *k*_iAno1_ and *k*_iNSCC_ (between 0 and 1 with step 0.05) the constraint 1.11 *k*_iAno1_ + *k*_iNSCC_ ⩽ 1.41 was identified for Step 1 of the optimisation problem. This excludes the combinations of parameter values which gave a non-physiological SW signal morphology.

**Figure 3.**
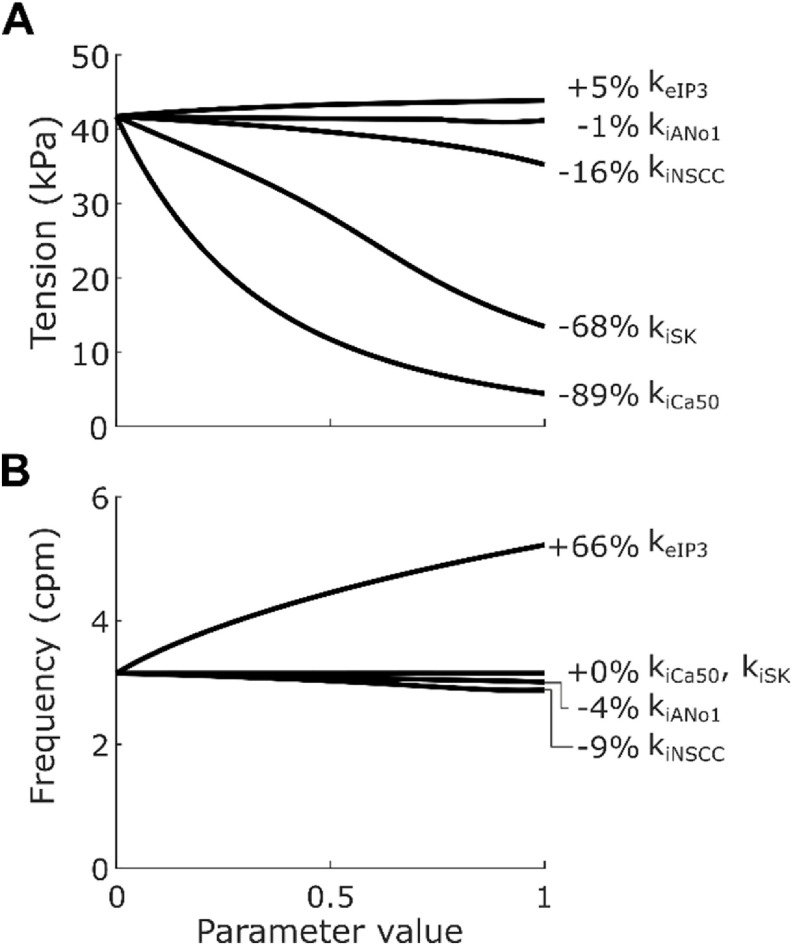
Plateau tension (A) and frequency (B) of phasic contractions when weighting parameters were individually varied from 0 to 1 in 0.1 increments, with f_i_ = 10 Hz and f_e_ = 10 Hz, non-varying constants set to 0, and non-linear scaling terms w_iICC_, w_iSMC_ and w_e_ set to 1. Metrics were calculated over 120 s of simulation time, with stimulation applied throughout this period (see section 2.5). When k_iAno1_, k_iNSCC_, k_iCa50_, k_iSK_, and k_eIP3_ were all set to 0 the frequency was 3.2 cpm and the plateau tension was 41.7 kPa, with the labelled percentages being the percentage change from this baseline when the varied parameter has value 1. Inhibition at ICC by Ano1 or NSCC was not sufficient to inhibit tension generation to the extent observed in experiments. Excitatory stimulation alone, via k_eIP3_, did not have a large effect on peak tension generation, but it did have a large effect on frequency.

Inspecting the change in plateau tension for varying weightings at maximal stimulation (figure 3(A)) showed that the Ano1 and NSCC inhibitory effector components, had a much smaller impact on tension compared to the effect of the SK channel (*k*_iSK_) and Ca^2+^ sensitivity (*k*_iCa50_). Additionally, excitatory stimulation in isolation had relatively little effect on ICC SW amplitude regardless of *k*_eIP3_. In contrast, excitatory stimulation via the IP_3_ effector component was the only component that had a large effect on SW frequency (figure 3(B)). The inhibitory effector components of Ano1 and NSCC both had small depressive effects on SW frequency, while other components had no impact on SW frequency.

The value of *k*_eIP3_ was based on the results reported by Forrest *et al* [36]. During *in vitro* electrical field stimulation at 5 Hz and 10 Hz the observed SW frequency was 5.2 cpm and 5.4 cpm, respectively. The similarity of the effect at different neural stimulation intensities was modelled by setting *p*_e_ to 5. The selected *k*_eIP3_ value was 1.0 to match the experimentally reported 62% increase in frequency attributable wholly to cholinergic electrical field stimulation at 5 Hz. On the other hand, at 10 Hz the response was not wholly attributable to cholinergic stimulation, and the value matching this response (0.82; figure 3(B)) was treated as the lower bound of the *k*_eIP3_ variable for sensitivity analysis.

**Figure 4.**
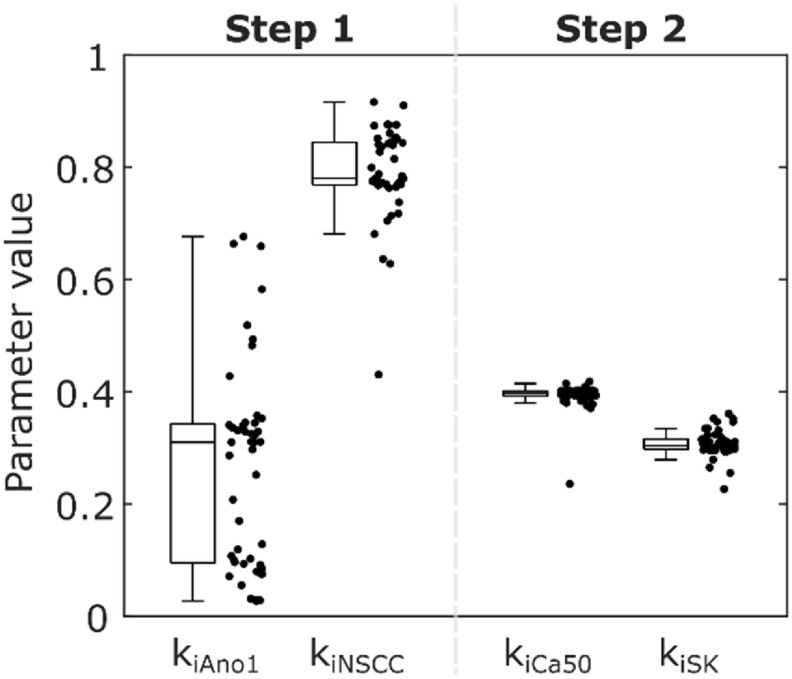
Results of the parameter optimisation procedure for effector component parameter values in two steps, showing a combined box plot and jittered scatter plot across the 48 initialisations. The selected parameter values are k_iAno1_ = 0.33, k_iNSCC_ = 0.77, k_iCa50_ = 0.40, k_iSK_ = 0.30.

Solving Step 1 of the parameter optimisation procedure yielded median values of 0.31, 0.78, and 3.16 for *k*_iAno1_, *k*_iNSCC_, and *p*_iICC_ respectively across the 48 initialisations, with two clusters of solutions, as shown in figure 4. To define the threshold between clusters in an unbiased manner a Gaussian kernel probability density estimate of the parameter value for *k*_iAno1_ was calculated with a half-width of 0.05. The parameter value with a minimal probability between the modal values was selected as the threshold (*k*_iAno1_ = 0.20). One of the clusters gave Ano1 almost no weighting (*k*_iAno1_ = 0.09, *k*_iNSCC_ = 0.84, *p*_iICC_ = 3.34) and the other gave Ano1 an effect, but the effect of NSCC was slightly diminished (*k*_iAno1_ = 0.33, *k*_iNSCC_ = 0.77, *p*_iICC_ = 3.15). The median (lower quartile, upper quartile) of the objective function (equation (28)) for the percentage change in ICC SW plateau amplitude was 2.66% (2.53%, 3.37%) overall, 2.45% (2.43%, 2.49%) for the low *k*_iAno1_ cluster, and 2.66% (2.65%, 2.66%) for the high *k*_iAno1_ cluster. Due to experimental evidence demonstrating the role of the Ano1 channel in IJPs the high *k*_iAno1_ cluster (*k*_iAno1_ = 0.33, *k*_iNSCC_ = 0.77, *p*_iICC_ = 3.15) was selected and carried forward to the next step [5, 42]. Solving Step 2 of the optimisation procedure resulted in median values of 0.40, 0.30, and 0.12 for *k*_iCa50_, *k*_iSK_, and *p*_iSMC_ respectively. The median (lower quartile, upper quartile) of the objective function (equation (28)) for the percentage change in phasic contraction plateau amplitude was 0.18% (0.16%, 0.20%). The large positive value of *p*_iICC_ indicates that the marginal effect of stimulation decreases with stimulation intensity for effector components in ICC. Conversely, the small value of *p*_iSMC_ indicates that a nearly linear shift in Ca_50_ and SK channel conductance modelled the response of SMC to inhibitory stimulation. Conducting Step 2 of the optimisation procedure with the low *k*_iAno1_ cluster values resulted in fitted similar values for *k*_iCa50_, *k*_iSK_, and *p*_iSMC_.

**Figure 5.**
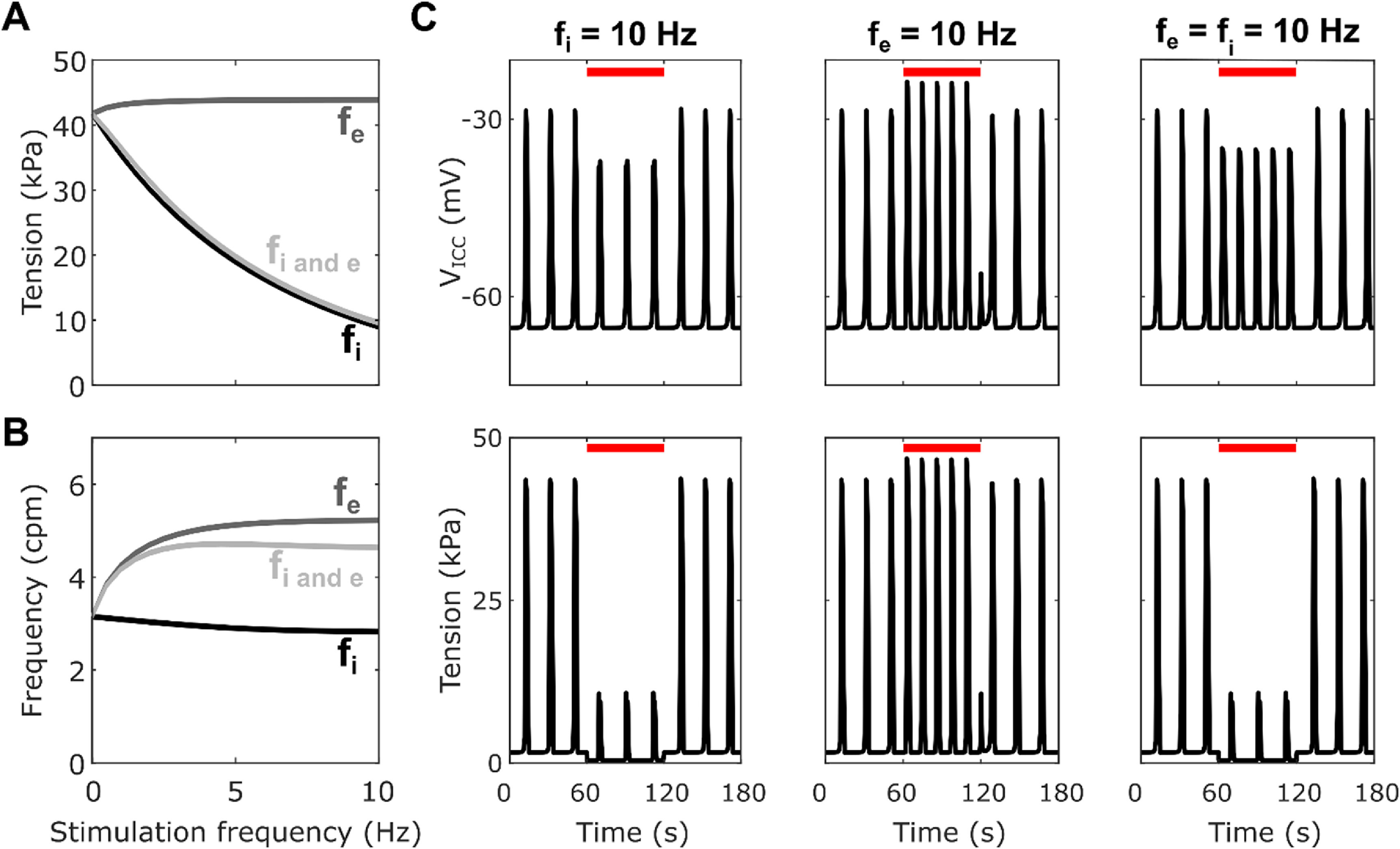
Plateau tension (A) and frequency (B) for varying f_i_ and/or f_e_ from 0 to 10 Hz with step 0.05 for the optimised values of the inhibitory effector components ([k_iAno1_, k_iNSCC_, k_iCa50_, k_iSK_] = [0.32, 0.78, 0.4, 0.30]). (C) Traces of the SW (top) and phasic contractions (bottom) for a stimulation duration of 60 s indicated by the red line with only inhibitory (f_i_ = 10 Hz), only excitatory; (f_e_ = 10 Hz), or both inhibitory and excitatory (f_i_ = f_e_ = 10 Hz) stimulation applied.

Inhibitory stimulation with the fitted parameters (figure 5) resulted in decreased plateau tension while excitatory stimulation resulted in a small increase in plateau tension. When both inhibitory and excitatory stimulation were applied equally there was an overall decrease in plateau amplitude, to a similar extent as that with just inhibitory stimulation (−77% at *f*_i_ = 10 Hz, *f*_e_ = 10 Hz vs −79% at *f*_i_ = 10 Hz, *f*_e_ = 0 Hz), indicating an overriding effect of the inhibitory stimulation. Frequency was greatly increased by excitatory stimulation, while inhibitory stimulation resulted in a small decrease in frequency (−10% at *f*_i_ = 10 Hz, *f*_e_ = 0 Hz). Simultaneous inhibitory and excitatory stimulation resulted in an overall increase in frequency, but this was a smaller increase than with only excitatory stimulation (+47% at *f*_i_ = *f*_e_ = 10 Hz vs +66% at *f*_i_ = 0 Hz, *f*_e_ = 10 Hz).

**Figure 6.**
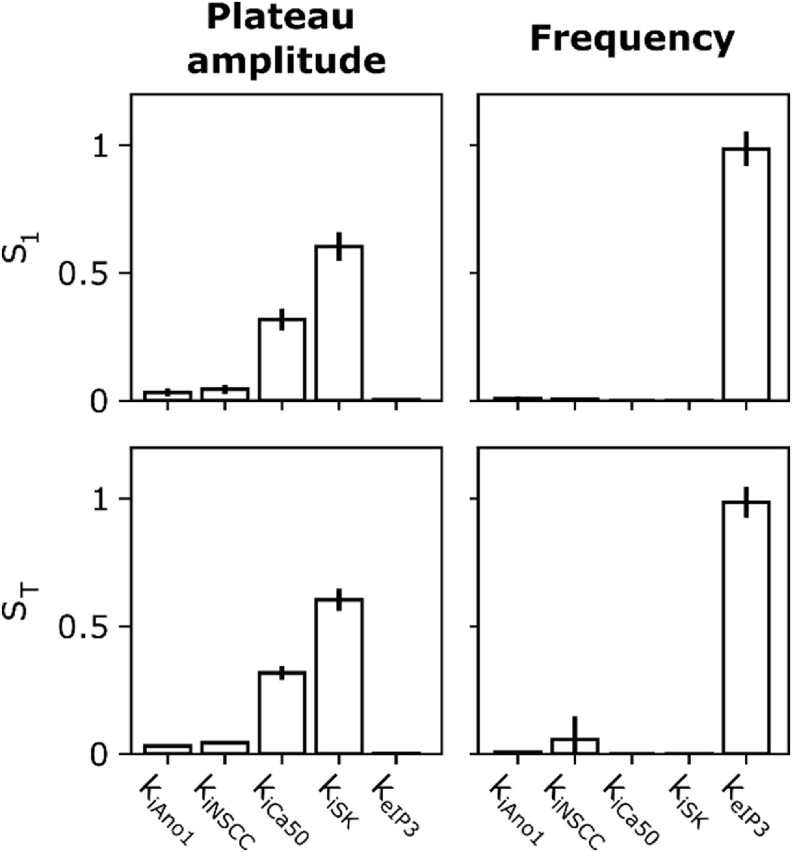
Parameter sensitivity analysis for neural regulation parameters. The first-order (top row) and total (bottom row) Sobol sensitivity indices are shown for the tension plateau amplitude (left column) and frequency (right column) metrics during the stimulation period. Vertical black lines atop bars indicate the 95% confidence interval for the sensitivity index.

Finally, the variance-based model sensitivity analysis (figure 6) notes the relative sensitivity of the model outputs to the fitted values of the effector component weights. This depends on the variance of the model outputs within the tested interval around the fitted parameter value. We see the variance in simulated tension plateau amplitude can be primarily attributed to perturbation of *k*_iSK_ (*S*_1_ = 0.60). Meanwhile the variance in simulated frequency was almost entirely attributable to perturbation of *k*_eIP3_ (*S*_1_ = 0.99). Simulating the model at the extreme values of these parameters in the tested sensitivity range with other parameters held at their fitted values shows that the absolute change in plateau tension amplitude decreased only 0.47% between for a 19% change in *k*_iSK_ from 0.293 to 0.343, while SW frequency increased only 4.9% between for an 18% change in the value of *k*_eIP3_ from 0.82 to 1.

## 4. Discussion

A novel model of gastric electrophysiology and motility incorporating neural regulation was developed. In conjunction, the effect of neural regulation on ICC SWs and phasic contractions was simulated. Key experimental literature was curated and used to inform and validate the model. The simulation results reinforce the relevance of SMC for inhibitory neural regulation. As a corollary, the model predicted a diminished role for Ano1 in influencing phasic contraction characteristics during neural stimulation, which contrasts with the clear role of Ano1 in generating slow IJPs. Simulated excitatory stimulation increased SW frequency but had a limited effect on phasic contraction amplitude.

The high value of the fitted parameter for SMC contractility, *k*_iCa50_, is consistent with observations in experimental literature where the change in SW amplitude was not commensurate with the much larger change in tension amplitude, measured simultaneously *in vitro* by Ozaki *et al* [47]. The sensitivity of the SMC contractile apparatus to Ca^2+^ is likely to be regulated by effects on myosin light chain phosphatase (MLCP) concentration via the cGMP signalling pathway [34]. A state-based model of active tension generation in gastric smooth muscle, such as that developed by Gajendiran and Buist [48], could be used to investigate this mechanism further.

Excitatory stimulation at SMC was not included in the model because experiments by Ozaki *et al* [49] showed that despite the presence of mechanisms that could transduce the effects of ACh on MLCP, no such relation was observed in the canine gastric antrum. This is supported by the lack of cholinergic excitatory junction potentials induced in the distal stomach of guinea pigs and rats [50, 51]. Furthermore, electrical field stimulation of mouse antral tissue deficient in ICC-IM did not yield the excitatory response seen in wild-type tissue both when stimulated for minutes, and when stimulated by short trains of stimulation [36, 52]. This indicates that the direct effect of the excitatory neural transmission is exerted at ICC and effects at SMC are indirect.

In the model, inhibitory stimulation at ICC had a relatively small impact on the amplitude and frequency of phasic contractions, compared to effects via SMC. However, experimentally observed changes in ICC SW characteristics would require changes at ICC, so the Ano1 and NSCC effector components were modelled. This is supported by the observation that IJPs involve Ano1, which is present in ICC but not SMC [53]. However, the cellular mechanisms generating IJPs need not be the same as those yielding a change in SW characteristics. The fitted parameters (figure 4(A)) suggest that of the two putative ICC effector components, *k*_iNSCC_ was relatively more important than *k*_iAno1_ in explaining the observed change in SW given inhibitory stimulation. The identity of channels contributing to the NSCC is yet to be identified in stomach tissue, though candidates include channels of the TRP family, some of which exhibit regulation by cGMP [25]. TRP family channels have been associated [24, 54] with ICC, and some evidence of TRPM7 has been found in human small intestinal ICC [55], though their role is controversial [24]. On the other hand, Ano1 channel inhibition, particularly in ICC-IM may play a role due to effects of NO on intracellular Ca^2+^ concentration. As discussed further by Suzuki *et al* [56], and more recently Sanders and Ward [5], the exact mechanism of NO mediated neural regulation that results in the effects on motility, rather than IJPs, is unclear.

The result observed in figure 5, where simultaneous inhibitory and excitatory stimulation resulted in an overall inhibitory effect is in consonance with observations made by Ozaki *et al* [57] using chemical stimulation with sodium nitroprusside and ACh, as well as the interaction of junction potentials in colonic tissue reported by Furness [58]. There was little change in the amplitude of phasic contractions during purely excitatory stimulation. This contrasts with experimental observations by Zhang *et al* [59], in which excitatory stimulation resulted in an increase in basal tone and phasic contraction amplitude. The basal tone did not increase during excitatory stimulation in the model. Taken together, these observations and previous studies suggest the inhibitory stimulation had an overriding effect on phasic contractions. This could have implications on the design of future closed-looped neurostimulator protocols [60].

Two key limitations of the developed model are related to the lumping of ICC subgroups, and the assumption of steady state effects. The populations of ICC in the stomach have been further sub-classified as myenteric (ICC-MY) or intramuscular (ICC-IM). Experimental evidence suggests that ICC-MY are responsible for the generation of regular SWs, while ICC-IM are neuroeffector cells interspersed between ICC-MY and SMCs [61]. Electrical coupling of ICC-MY and ICC-IM to SMC is responsible for initiating the influx of Ca^2+^ which leads to active tension generation. ICC-IM were not explicitly modelled here, but the electrical coupling input to SMC models both the pacemaking and neurotransduction aspects of ICC function. Explicitly modelling ICC-IM and ICC-MY as separate but coupled cells would improve our ability to distinguish the cellular mechanisms required for gastric neural regulation. Furthermore, the intricate structural arrangements and biophysical relationships of different cell sub-classes of ICC and PDGFR*α* ^+^ cells would need to be considered across extended spatial scales. This is a challenging task that requires sophisticated whole-mount imaging of transgenic animals or specific labelling of ICC sub-types accompanied by investigations of specific channels and channel control mechanisms [62].

Additionally, a candidate for the incorporation of further detail into the model is the time-course of neural regulation. In the present work, the interaction between motor neurons and gastric motility were simulated by modelling cellular mechanisms in ICC and SMC with a frequency-dependent, steady-state motor neuron stimulation input. The experimental data against which the model was evaluated used protocols involving either electrical or chemical stimulation at this time scale [36, 37, 57, 59]. However, some post-junctional effects of neural regulation, such as junction potentials, occur at a much shorter time scale. The stimulation intensity terms, *f*_i_ and *f*_e_, used in the present model assume that prolonged stimulation at a given frequency results in a steady-state of functional response. In biological settings, this intensity is time-varying at a shorter time-scale and determined by neurotransmitter release characteristics including release frequency, release volume, and the number of endings as well as the physical transport of neurotransmitters across the junction. Consideration of shorter time scales would refine this aspect of the model and could help to further distinguish the cellular mechanisms responsible for post-junctional effects. To this end, it will be valuable in future to obtain sufficient experimental data to be able to model the transient aspects of transmission and events occurring post-stimulation.

The experimental parallel of this model lies in the data used for fitting, wherein electrical field stimulation was applied for many minutes at a fixed frequency, with pulse widths of 0.1 ms [36, 37]. Effects at a longer time-scale, as modelled in the present work, are particularly valuable for extension into whole organ modelling applications related to clinical applications of neuromodulation. Direct electrical pacing stimulation of the porcine stomach has shown that spatiotemporal entrainment of gastric bioelectrical activity at the organ-scale occurs over many SW cycles, on the order of minutes [63].

Simulation of neural regulation at the whole-organ level is particularly relevant in the context of peripheral nerve stimulation research. Models of intestinal motility at the organ level have been used to successfully simulate the transit of colonic and small intestinal luminal contents in conjunction with autonomic nervous system regulation [21, 64]. The neurons that influence gastric function operate as components of reflex circuits and work in conjunction with other influences, such as muscle stretch and hormones [1]. A comprehensive model of the gut-brain axis would be ideal but not tractable in the present study due to its complexity and insufficient understanding of its mechanisms.

Although pathological states were outside the scope of the present work, we believe that our model will provide a basis to investigate pathological changes in gastric electrophysiology and function caused by perturbations of specific mechanisms in our model. Future work bridging the gap between the peripheral nerves, such as the vagus nerve, and enteric neurons will be valuable for applying this model in the context of peripheral nerve stimulation. Particular phenomena, such as vago-vagal reflexes, operate through these pathways and control gastric function unrelated to the phasic contractions of the distal stomach [2]. The vagus nerve influences the gastric portion of the ENS, and recent interest in vagus nerve stimulation as a clinical therapy for a variety of peripheral organ ailments raises the question of how gastric motility is affected by vagus nerve stimulation [15, 65]. Whole organ modelling, incorporating regionally divergent structural and functional properties of the stomach [66, 67], would result in tangible clinical outcomes stemming from the developed mathematical model.

## 5. Conclusion

A novel mathematical model of enteric neural regulation of gastric motility was developed and used to determine the mechanisms through which enteric neural influence is exerted. The parameter optimisation procedure predicted that the inhibition of SMC Ca^2+^ sensitivity is an essential part of the functional inhibitory response in this model. Additionally, the inhibition of Ano1 alone during inhibitory stimulation was unable to explain experimentally observed effects on ICC SW amplitude. Furthermore, it was determined that excitatory stimulation affecting ICC alone can explain experimentally observed changes in frequency but not in changes in amplitude. This model is primed for use in further investigations which extend to include vagus nerve influence on gastric ENS regulation and downstream effects on gastric motility at the tissue to whole organ scale.

## Data Availability

The data that support the findings of this study are openly available at the following URL/DOI: 10.6084/m9.figshare.23624622 [68]. Additionally, an interactive model simulation is available on o^2^S^2^PARC: https://osparc.io/study/1170826c-9a64-11ee-8b3c-02420a0b1983.
